# High-Mobility Group Box 1 Mediates Fibroblast Activity via RAGE-MAPK and NF-κB Signaling in Keloid Scar Formation

**DOI:** 10.3390/ijms19010076

**Published:** 2017-12-28

**Authors:** Jihee Kim, Jong-Chul Park, Mi Hee Lee, Chae Eun Yang, Ju Hee Lee, Won Jai Lee

**Affiliations:** 1 Department of Dermatology & Cutaneous Biology Research Institute, Yonsei University College of Medicine, Seoul 03722, Korea; mygirljihee@yuhs.ac; 2 Department of Medical Engineering, Yonsei University College of Medicine, Seoul 03722, Korea; parkjc@yuhs.ac (J.-C.P.); LEEMH1541@yuhs.ac (M.H.L.); 3 Department of Plastic and Reconstructive Surgery, Institute for Human Tissue Restoration, Yonsei University College of Medicine, Seoul 03722, Korea; CHENIYA@yuhs.ac

**Keywords:** keloid, hypertrophic scar, fibroblast, HMGB1

## Abstract

Emerging studies have revealed the involvement of high-mobility group box 1 (HMGB1) in systemic fibrotic diseases, yet its role in the cutaneous scarring process has not yet been investigated. We hypothesized that HMGB1 may promote fibroblast activity to cause abnormal cutaneous scarring. In vitro wound healing assay with normal and keloid fibroblasts demonstrated that HMGB1 administration promoted the migration of both fibroblasts with increased speed and a greater traveling distance. Treatment of the HMGB1 inhibitor glycyrrhizic acid (GA) showed an opposing effect on both activities. To analyze the downstream mechanism, the protein levels of extracellular signal-regulated kinase (ERK) 1/2, protein kinase B (AKT), and nuclear factor kappa-light-chain-enhancer of activated B cells (NF-κB) were measured by western blot analysis. HMGB1 increased the expression levels of ERK1/2, AKT, and NF-κB compared to the control, which was suppressed by GA. HMGB1 promoted both normal and keloid fibroblasts migration to a degree equivalent to that achieved with TGF-β. We concluded that HMGB1 activates fibroblasts via the receptor for advanced glycation end product (RAGE)—mitogen-activated protein kinases (MAPK) and NF-κB interaction signaling pathways. Further knowledge of the relationship of HMGB1 with skin fibrosis may lead to a promising clinical approach to manage abnormal scarring.

## 1. Introduction

Keloids, or hypertrophic scars, are formed by the excessive accumulation of extracellular matrix (ECM) substrates caused by abnormal wound healing processes. Recently, numerous hypotheses regarding the cause of keloids have been proposed, including aberrant cellular responses to mechanical strains, upregulation of growth factors and inflammatory cytokines, the epithelial-mesenchymal transition (EMT), and regulation of cellular apoptosis [1,2,3,4]. Abnormal fibroblast activity is a histopathological hallmark for keloid pathogenesis, as it results in excessive synthesis of ECM components, especially collagen. Among the various signaling molecules involved in keloid pathogenesis, transforming growth factor-beta (TGF-β) is a crucial mediator. In keloid tissues and fibroblasts, proteins involved in TGF-β signal transduction are overexpressed. TGF-β promotes signaling via the Smad and Wnt-dependent pathways to enhance cell proliferation, resulting in excessive ECM production and deposition [5,6].

High-mobility group box 1 (HMGB1) is a member of the HMGB protein family, which was originally identified as a nuclear non-histone DNA-binding protein that acts as a critical co-factor of somatic cell transcriptional regulation [7]. It plays roles in a wide variety of processes, including inflammation, immune responses, apoptosis, and responses to injury [8,9]. As an intracellular transcription factor, HMGB1 modulates cellular transcription, recombination, replication, and repair [10]. Extracellular HMGB1 is released and stimulated by necrotic, damaged, or inflammatory cells after tissue injury and acts as a potent inflammatory cytokine. Extracellular HMGB1 also functions as a chemo-attractant; these functions are mediated by interactions with cell-surface receptors such as Toll-like receptors (TLRs) and the receptor for advanced glycation end product (RAGE). Exogenous HMGB1 also pairs with the chemokine C-X-C motif chemokine 12 (CXCL12), which binds to the CXCR4 chemokine receptor [11]. These receptors activate multiple intracellular signaling pathways, including mitogen-activated protein kinases (MAPKs), extracellular signal-regulated kinase (ERK) 1/2, and phosphatidylinositol-4,5-bisphosphate 3-kinase (PI3K)/protein kinase B (AKT), which are known to be related to wound healing processes. In keloid pathogenesis, activation of the PI3K/AKT signaling pathway is known to be responsible for stimulating collagen synthesis.

The release of HMGB1 after tissue injury and the subsequent onset of fibrosis has been reported in multiple organs. Emerging studies have revealed that HMGB1 is strongly associated with fibrotic diseases in the liver, renal, lung, and myocardial tissue, while the inhibition of HMGB1-related signaling pathways was shown to prevent fibrosis in experimental animal models [12,13,14,15]. During wound healing, HMGB1 is thought to influence the healing process as a pro-fibrotic element. Recent studies have demonstrated that HMGB1 binds to cell-surface receptors such as RAGE and TLR2/4 in keratinocytes and fibroblasts [16]. When administered to early embryonic murine skin, which normally heals without scarring, HMGB1 induced scarring and fibrosis [17]. However, the roles of HMGB1 in wound healing and associated cutaneous scarring are currently unclear.

Although the role of HMGB1 in wound healing has been investigated, no studies to date have evaluated its role in cutaneous scarring and keloid development. We hypothesized that HMGB1 may promote fibroblast activity, ultimately causing abnormal cutaneous scarring. Therefore, in this study, we investigated the motility and migration activity of fibroblasts in response to HMGB1 and its inhibitor glycyrrhizic acid (GA). Additionally, to study the underlying molecular mechanism upon HMGB1 activity, we analyzed the expression of RAGE and MAPK signaling pathway molecules.

## 2. Results

### 2.1. HMGB1 Administration Promoted Fibroblast Migration

**Figure 1:**
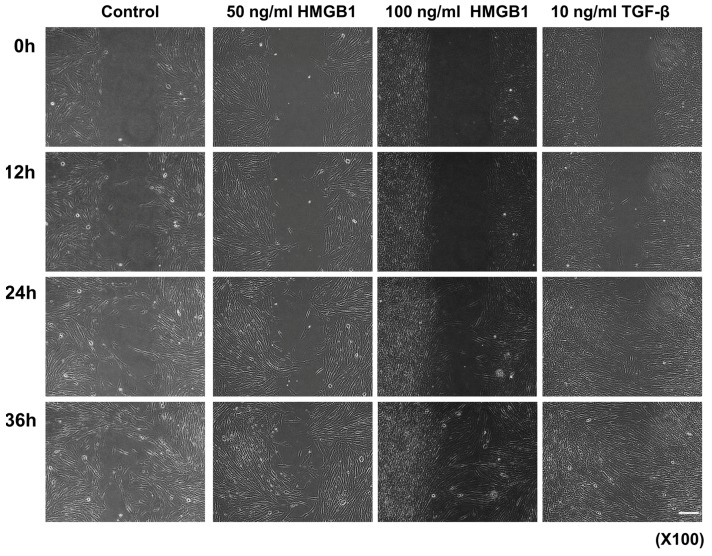
In vitro wound healing assay and cell-tracking system for cell migration. Scale bar: 50 μm. Recovery of the denuded space was completed after 36 h in normal fibroblasts. However, the wound area on the 50 ng/mL and 100 ng/mL High-mobility group box 1 (HMGB1)-treated and 10 ng/mL TGF-β-treated groups showed more rapid closure than the untreated control group.

Enhanced migration and invasion of normal fibroblasts are critical factors for the development of keloid diseases. Therefore, we investigated whether HMGB1 would affect the behavior of normal fibroblasts in vitro using a wound healing assay model. TGF-β was administered to compare its effect with HMGB1. The initial wound width of untreated normal fibroblasts was approximately 500 μm; after 36 h, migrating cells almost completely covered the denuded area. However, the wound area on the 50 ng/mL and 100 ng/mL HMGB1-treated and 10 ng/mL TGF-β-treated groups showed rapid closure compared to the untreated control group. Furthermore, more than half of the wound area in the untreated control group remained uncovered after 24-h incubation (Figure 1).

### 2.2. HMGB1 Administration Increased the Migration Speed and Distance Traveled in Normal Fibroblasts

**Figure 2:**
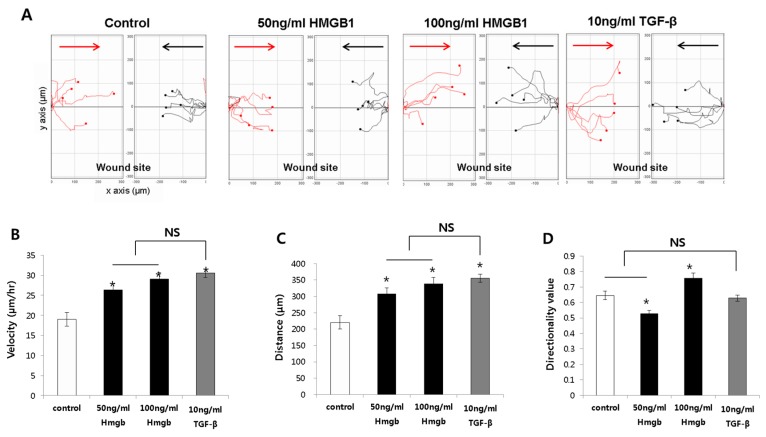
The cell tracking system for fibroblast migration was analyzed using cells selected from both sides of the wound, cells were tracked toward the site of wound insert after cultivation for 24 h; black, cells toward the midline from the left edge; red, cells toward the midline from the right edge (**A**); The average migration speed (**B**) and average accumulated distance (**C**) traveled after treatment with HMGB1 or TGF-β; (**D**) Directionality values in response to HMGB1 or TGF-β treatment. All results are shown as mean ± SD (* *p* < 0.05).

Cells from the control, HMGB1-treated (50 and 100 ng/mL), and TGF-β-treated (10 ng/mL) groups were selected from both sides of the wound, and the cell migration speed, distance, and directionality were analyzed (Figure 2A).

The average migration velocities of the 50 ng/mL and 100 ng/mL HMGB1-treated normal fibroblast were 26.31 ± 4.00 μm/h and 29.06 ± 4.33 μm/h, respectively. This was significantly higher than the speed of the untreated normal fibroblasts (19 ± 4.25 μm/h; *p* < 0.05; Figure 2B). The average migration speed of the TGF-β-treated group was higher than that of the control group (30.51 ± 2.78 μm/h). However, there was no significant difference between the HMGB1-treated group and the TGF-β-treated group (Figure 2B).

The total distances traveled by the 50 ng/mL and 100 ng/mL HMGB1-treated normal fibroblasts were 307.60 ± 47.45 μm and 338.70 ± 50.90 μm, respectively, which was significantly higher than that of the untreated group (220.60 ± 49.26 μm; *p* < 0.05; Figure 2C). Furthermore, the distance traveled by the 10 ng/mL TGF-β-treated group was greater than that of the untreated normal fibroblasts (355.70 ± 33.35 μm; *p* < 0.05), but there was no significant difference compared to the HMGB1-treated groups. The total distance traveled by the 50 ng/mL (297.80 ± 61.12 μm) and 100 ng/mL (285.30 ± 28.45 μm) HMGB1-treated groups was greater than that of the untreated group (219.20 ± 30.06 μm; *p* < 0.05; Figure 2C). In addition, the distance traveled by the 10 ng/mL TGF-β-treated group (323.20 ± 42.71 μm) was greater than that of the untreated group (Figure 2C).

Next, we investigated the directionality values of normal fibroblast cells in response to HMGB1 treatment. The directionality value indicates the potential of the cell to migrate closer to the midline when initiating vertically from the wound edge. The results for the control, 50 ng/mL, 100 ng/mL HMGB1-treated, and 10 ng/mL TGF-β-treated normal fibroblasts were 0.65 ± 0.08, 0.52 ± 0.06, 0.76 ± 0.09, and 0.69 ± 0.05, respectively. As shown in Figure 2D, only the 100 ng/mL HMGB1-treated group showed a significant increase in directionality compared to the control (*p* < 0.05). These results indicate that fibroblasts showed greater directionality toward the wound center, displayed higher migration velocities, and traveled longer distances after HMGB1 treatment. Similar results were noted in TGF-β-treated cells.

### 2.3. Treatment with Glycyrrhizic Acid (GA), an HMGB1 Inhibitor, Decreased the Migration Speed and Distance Traveled in Normal Fibroblasts

**Figure 3:**
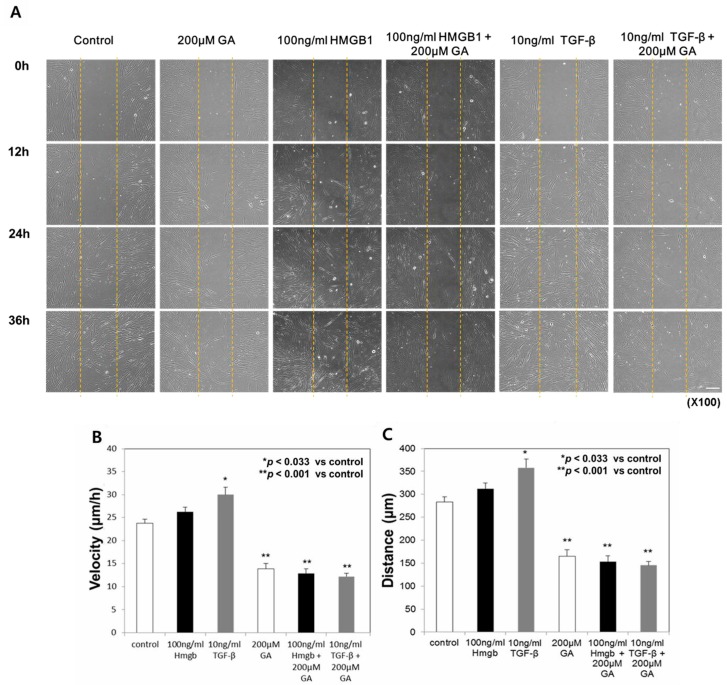
Fibroblast migration was delayed by glycyrrhizic acid (GA) treatment. Scale bar: 50 μm (**A**) GA induced a decrease in the average migration speed (**B**) and the average distance (**C**) traveled of normal fibroblasts. All results are shown as mean ± SD (* *p* < 0.033, ** *p* < 0.001).

The above results demonstrated that HMGB1 induces an increase in the cell migration speed and distance of normal fibroblasts, as in TGF-β-treated cells. We added GA, a known inhibitor of HMGB1, to evaluate its effect on HMGB1-induced fibroblast migration. In all three groups treated with 200 μM GA, fibroblast migration was considerably reduced. Most of the area remained uncovered after 36-h incubation, although cells at the margin showed forward movement (Figure 3A).

The average migration speed of the 100 ng/mL HMGB1- and 10 ng/mL TGF-β-treated normal fibroblasts was 26.11 ± 3.27 μm/h and 29.99 ± 5.18 μm/h, respectively. The TGF-β-treated group showed significant increase compared to that of untreated normal fibroblasts (23.76 ± 2.92 μm/h; *p* < 0.033). Treatment with 100 μM GA did not significantly affect the fibroblast migration speed or distance. However, treatment with 200 μM GA induced a significant decrease in migration speed in both the HMGB1- and TGF-β-treated groups (12.84 ± 3.37 μm/h and 12.18 ± 2.23 μm/h, respectively; *p* < 0.001, Figure 3B).

The total distances traveled by the 100 ng/mL HMGB1- and 10 ng/mL TGF-β-treated normal fibroblasts were 312.41 ± 39.01 μm and 357.52 ± 61.78 μm, respectively, greater than that of the untreated group (283.23 ± 34.86 μm; *p* < 0.05). After treatment with 200 μM GA, the distance was significantly reduced in both the HMGB1- and TGF-β-treated groups (153.06 ± 40.21 μm and 145.19 ± 26.56 μm, respectively; *p* < 0.001, Figure 3C).

### 2.4. GA Treatment Decreased the Migration Speed and Distance Traveled in Keloid Fibroblasts

**Figure 4:**
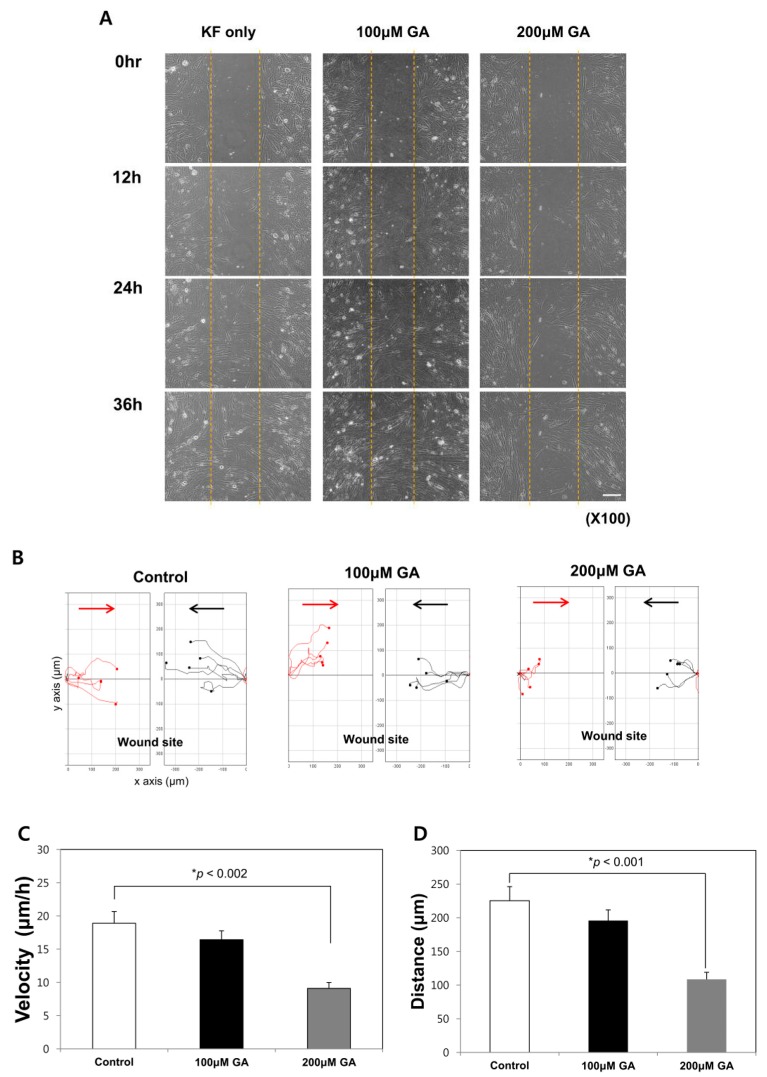
Glycyrrhizic acid (GA) inhibited keloid fibroblast activity. Scale bar: 50 μm. (**A**) The in vitro wound healing assay showed decreased keloid fibroblast migration after GA treatment; (**B**) The cell tracking system for fibroblast migration was analyzed using cells selected from both sides of the wound, keloid fibroblasts were tracked toward the site of wound insert after cultivation for 24 h; black, cells toward the midline from the left edge; red, cells toward the midline from the right edge. GA-induced decreases in the average migration speed (**C**) and the average distance (**D**) traveled of keloid fibroblasts. All results are shown as mean ± SD (* *p* < 0.05).

In vitro migration assays were performed with keloid fibroblasts. Cell migration was initiated after 12 h, and the denuded area was almost completely covered by migrating cells after 36 h. When GA was administered, wound recovery was inhibited in a dose-dependent manner. After treatment with 200 μM GA, more than half of the area remained uncovered, even after 24-h incubation (Figure 4A,B).

The average migration speed of 200 μM GA-treated keloid fibroblasts was 9.11 ± 2.78 μm/h, which was significantly lower than that of untreated keloid fibroblasts (18.91 ± 5.56 μm/h; *p* < 0.01, Figure 4C). The average total distance traveled by untreated keloid fibroblasts was 225.37 ± 66.28 μm. After treatment with significantly decreased following treatment with 200 μM GA (*p* < 0.001, Figure 4D). The directionality values for the untreated and 200 μM GA-treated groups were 0.68 ± 0.21 and 0.68 ± 0.11, respectively, indicating no significant change.

### 2.5. HMGB1-Induced ERK1/2, AKT, and NF-κB Protein Expression was Suppressed by GA Treatment in Human Normal Fibroblasts

When HMGB1 is released by damaged or necrotic cells, NF-κB activation is required to promote cell migration. When HMGB1 binds to TLR2/4, the activated TLR triggers activation of the PI3K/AKT signaling cascade. Therefore, we assessed whether HMGB1-induced cell migration may involve the intracellular signaling pathways of ERK1/2, AKT and NF-κB, and whether GA treatment would inhibit these pathways.

**Figure 5:**
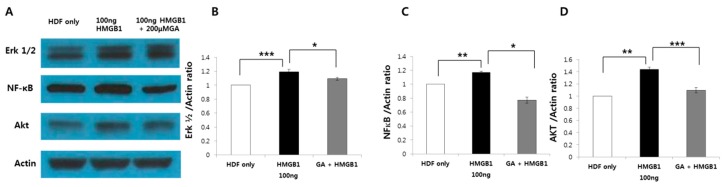
HMGB1 (100 ng/mL) increased the expression levels of ERK1/2, AKT, and NF-κB. (**A**) HMGB1-induced expression of internal signaling cascade molecules such as ERK1/2 (**B**); NF-κB (**C**); and AKT (**D**) was significantly decreased by treatment with 200 μM GA (*** *p* < 0.001; ** *p* < 0.01; * *p* < 0.05).

**Figure 6:**
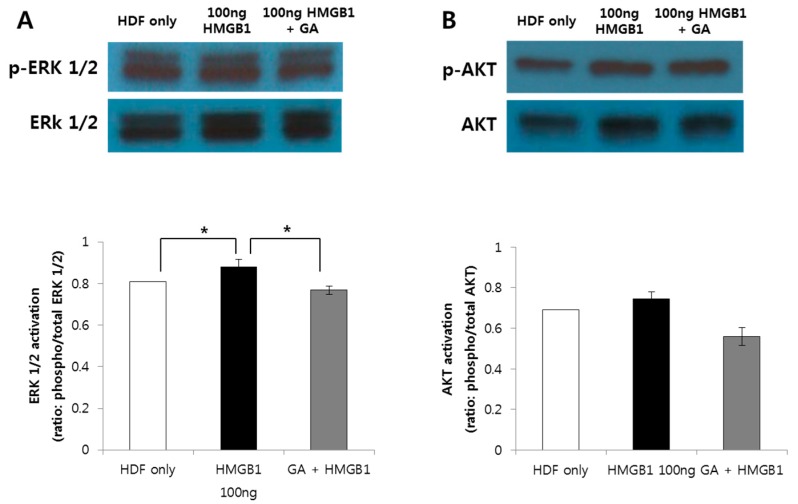
HMGB1 (100 ng/mL) increased the expression levels of phosphorylated form of ERK1/2 (p-ERK) and AKT (p-AKT). Assessment of both phosphorylated and total ERK1/2 (**A**) and AKT (**B**) was done to detect the activation of signaling cascade. The graph shows the ratio between the optical density of the bands of the phosphorylated form and the bands of the corresponding total protein. HMGB1-induced increase of p-ERK1/2 and p-AKT was decreased by treatment with 200 μM GA and the change was statistically significant in p-ERK1/2 (* *p* < 0.05).

The protein levels of ERK1/2, AKT, and NF-κB were measured by western blot analysis. HMGB1 (100 ng/mL) treatment significantly increased the expression levels of ERK1/2, AKT, and NF-κB compared to those of the control group (*p* < 0.001; *p* < 0.01; *p* < 0.05; Figure 5A). In addition, the HMGB1-induced expression of all three proteins was significantly decreased by treatment with 200 μM GA (Figure 5B). Furthermore, challenging HMGB1 on HDF also increased ERK1/2 and AKT phosphorylation and its inhibition attenuated the activation of ERK1/2, AKT signaling molecules (Figure 6).

## 3. Discussion

HMGB1 is normally found in the nucleus, and functions as an intranuclear architectural DNA-binding protein that is associated with transcription factors. When cell damage occurs, HMGB1 is translocated to the cytoplasm and released by the cell to act as a multifunctional cytokine with roles in infection, organ dysfunction, inflammation, and immune responses [17,18]. Recent studies of wound healing have demonstrated that HMGB1 binds to cell-surface receptors and acts as a promoter of wound closure through its chemotactic effect on skin fibroblasts and keratinocytes [16,17]. HMGB1 levels were found to be decreased in diabetic human and mouse skin, which may account for the altered wound healing in diabetic patients [16].

Fibroblast dysfunction and associated ECM deposition is a histopathological hallmark in keloid pathogenesis. Unlike normal fibroblasts, keloid fibroblasts possess tumor-like properties, showing excessive proliferation and invasion of surrounding tissues [19,20]. Enhanced migration and invasion of normal fibroblasts are critical factors for the development of keloids [21].

The present study was designed to evaluate the role of HMGB1 in the development of keloids by conducting an in vitro assay to uncover its mechanism of action in normal and keloid fibroblasts. The results of this study demonstrated that HMGB1 alters the behavior of both normal and keloid fibroblasts. Treatment with HMGB1 promoted an increase in the migration of both normal and keloid fibroblasts to a degree equivalent to that achieved by treatment with TGF-β. Subsequent in vitro wound healing assays demonstrated an increase in the migration speed and distance traveled in both cell lines. Furthermore, HMGB1-induced increases in the motility of normal and keloid fibroblasts were significantly inhibited by treatment with GA.

There are several mechanisms by which HMGB1 promotes the fibroblast proliferation associated with keloid development. In keloid tissues, fibroblast proliferation is influenced by epithelial-mesenchymal interactions between the surrounding keratinocytes and fibroblasts [22]. In a previous study, we demonstrated that the EMT-like transition via Wnt3a activation substantially contributes to collagen accumulation during the development of keloids [23]. Emerging experimental evidence indicates that HMGB1 also plays an important role in EMT. HMGB1-induced EMT has been identified in colorectal and gastric cancers, and is activated by the RAGE/NF-κB pathways [24,25]. In estrogen-mediated wound healing, HMGB1 has been shown to greatly accelerate keratinocyte migration, and its knockdown blocked estrogen-induced keratinocyte migration [26]. Cardiac fibroblasts actively secrete HMGB1 in response to mechanical stress or inflammation, which leads to cardiac collagen deposition via the PKCβ/ERK1/2 signaling pathway [27,28]. In human airway epithelial wound closure, HMGB1 has been shown to induce TLR4- and RAGE-mediated wound closure, ECM protein and receptor expression, and intracellular signaling. In particular, the similarities between HMGB1 and TGF-β in wound closure in human bronchial models suggest that changes associated with EMT may occur as part of the repair process upon exposure to HMGB1 [13,29].

To evaluate the presence of HMGB1-mediated EMT in normal fibroblasts, we investigated the expression of downstream molecules involved in HMGB1 signaling. Among its multiple cellular receptors, HMGB1 has been shown to interact with TLR2/4 and RAGE in injury and inflammation models to trigger subsequent inflammatory signaling [30,31,32,33,34]. Specifically, HMGB1 induces an increase in the expression levels of ERK1/2, AKT, and NF-κB. Moreover, these increases were significantly decreased in the presence of an HMGB1 inhibitor. Considering that ERK1/2 is a downstream molecule of RAGE, and that AKT is secreted via TLR2/4 activation and the PI3K cascade, our results support the current hypothesis that extracellular HMGB1 acts on normal fibroblasts by binding to both RAGE and TLR2/4. Activation of the ERK and PI3K/AKT pathways is associated with the proliferation of keloid fibroblasts, along with keratinocytes, and eventually leads to excessive collagen accumulation [35]. In keloid tissue, simultaneous activation of the ERK and PI3K/AKT pathways is important for the production of collagen and other ECM components [36].

TGF-β/Smad signaling has long been considered a pivotal fibrogenic factor in abnormal wound healing. Subsequently, anti-fibrotic strategies based on the blockade or elimination of TGF-β signaling emerged as an important pharmacological target for treating keloids [37,38,39]. Although there are currently no effective therapeutic interventions for keloids, small-molecule inhibitors targeting HMGB1 or its receptors based on silencing HMGB1, RAGE, or TLRs have proven successful in reducing the severity of symptoms in various experimental models of fibrotic diseases [13,14,25,40,41].

Glycyrrhizin and its metabolites have been reported to suppress tissue fibrosis by targeting TGF-β and other fibrosis-related pathological signaling molecules [42,43]. In the present study, administration of the HMGB1 inhibitor GA resulted in decreased migration and motility in HMGB1- and TGF-β treated normal fibroblasts. In addition, treatment with GA caused decreased migratory property of normal fibroblasts. It is well demonstrated that inflammation during the wound healing period induces transition of normal fibroblast to keloid fibroblasts [44]. GA is known to inhibit HMGB1 activity by inhibiting its pro-inflammatory activity [45]. When applied to keloid fibroblasts, cellular activity represented by migration speed and distance was significantly impeded. This effect of GA can be attributed to its inhibitory effects on both the RAGE and TLR2/4-activated signaling of HMGB1. In a previous study, we demonstrated that HMGB1 induces the expression of EMT-associated proteins in normal fibroblasts; thus, we hypothesized that inhibiting HMGB1 activity or its related signals could be beneficial for treating or even preventing keloids. Theoretically, inhibition of HMGB1 can suppress EMT and the activation of associated pro-fibrotic cytokines. However, because HMGB1 interacts with multiple signaling systems and undergoes dynamic post-translation modifications, it is important to optimize strategies for blocking its abnormal activation.

Currently available studies have already demonstrated that HMGB1 promotes wound healing. Our study further demonstrates that HMGB1 may induce the fibroproliferative properties associated with excessive collagen accumulation, as observed in keloid tissue. Unlike previous studies, which showed increased or decreased expression levels of HMGB1 under certain conditions, our study focused on the molecular signaling pathways associated with keloid development. We suggest that after skin injury, extracellular HMGB1 induces RAGE- and TLR2/4-mediated fibroblast activation. This may provoke the activation of a TGF-β-like profibrotic effect via the ERK1/2 and PI3K/AKT pathways, which would eventually lead to EMT-like changes, causing abnormal ECM accumulation in keloid tissues.

## 4. Materials and Methods

### 4.1. Keloid Tissues, Keloid-Derived Fibroblasts, and Human Dermal Fibroblast Cell Culture

Keloid tissues were collected from patients with active-stage keloid (*n* = 5) after obtaining informed consent according to the protocol approved by the Yonsei University College of Medicine Institutional Review Board (IRB No. 4-2015-0228, 29 JUL 2015) All experiments involving humans were performed in accordance with the Declaration of Helsinki. Human normal dermal fibroblasts were obtained from the American Type Culture Collection (Manassas, VA, USA). Separated cells were cultured in Dulbecco’s modified Eagle’s medium (Gibco, Grand Island, NY, USA) supplemented with 10% heat-inactivated fetal bovine serum, penicillin (30 U/mL), streptomycin (300 μg/mL), and actinomycin. The culture medium was replaced every 2–3 days.

### 4.2. In Vitro Wound Healing Assay and Cell Tracking System for Cell Migration

An in vitro wound healing model was established using silicon culture inserts (Ibidi, Munich, Germany) with two individual wells for cell seeding. Each insert was placed in a culture dish; 1 × 10^4^ normal or keloid fibroblasts were plated in each well and grown to form a confluent and homogeneous layer. Twenty-four hours after cell seeding, the culture insert was removed to form an approximately 500-μm-wide cell-free “wound” area for observation. Cells were treated with 50 or 100 ng/mL HMGB1 and 10 ng/mL TGF-β in media. Wound healing by cell migration over time was measured under a light microscope (IX-70, Olympus, Tokyo, Japan).

To track the cells, they were treated with HMGB1 and TGF-β in media, cultured in a mini-incubator (Live Cell Instrument, Seoul, Korea), visualized by light microscopy, and cell images were recorded every 5 min for 36 h by a charge-coupled device camera (Electric Biomedical Co., Ltd., Osaka, Japan) attached to an inverted microscope (Olympus). Captured images were analyzed using Image J software, version 1.48J. Image analysis was carried out by manual tracking, as well as the chemotaxis and migration tool plug-in V1.01 (Ibidi, Planegg, Germany). Cells were selected from both sides of the wound. We obtained the datasets of XY coordinates by using manual tracking, then these datasets were imported into chemotaxis plug-in. These datasets were then imported into the chemotaxis and migration tool plug-in, which computed the cell migration speed and directionality and plotted the cell migration pathway.

The migration speed was calculated as an accumulated distance of the cell divided by time. The migration distance of the cell was defined as the straight-line distance along the *y* axis between the start position and the end position of cell divided by accumulated distance. For each experiment, cells were randomly selected along each edge of the wound. Cells undergoing division, death or migration outside the field of the view were excluded from the analysis.

We added GA, a known inhibitor of HMGB1, to evaluate its effect on HMGB1-induced fibroblast migration. GA (200 μM) was administered to the 100 ng/mL HMGB1- and 10 ng/mL TGF-β-treated normal fibroblasts to analyze its effects. Additionally, 100 μM and 200 μM GA was treated to keloid fibroblasts.

### 4.3. Western Blotting Analysis

Normal and keloid fibroblasts were grown to 70% confluence in 100 × 20-mm cell culture dishes. Cultured keloid fibroblasts were exposed to HMGB1 (100 ng/mL) for 48 h. The protein (20 μg) was subjected to 10% sodium dodecyl sulfate-polyacrylamide gel electrophoresis and electrophoretically transferred onto a polyvinylidene fluoride membrane (Millipore, Billerica, MA, USA). The membranes were blocked with blocking buffer for 1 h and incubated with primary antibodies against AKT (1:1000, rabbit polyclonal; Cell Signaling Technology, Danvers, MA, USA), p-AKT (Cell Signaling Technology), ERK 1/2 (Cell Signaling Technology), p-ERK 1/2 (Cell Signaling Technology) and actin (1:5000, mouse monoclonal; Sigma-Aldrich, St. Louis, MO, USA) before incubating overnight at 4 °C. Secondary antibodies against horseradish peroxidase-conjugated rabbit antibody (1:2000, Santa Cruz Biotechnology, Santa Cruz, CA, USA) and mouse antibody (1:2000, Santa Cruz Biotechnology) were then added and the membrane was incubated for another 2 h at room temperature. After incubation with secondary antibodies, the membrane blot was developed using an electrochemiluminescence blotting system (Amersham Pharmacia Biotech, Piscataway, NJ, USA) according to the manufacturer’s instructions; the densities of the bands on the developed film were analyzed using Image J software. Protein expression levels were normalized to those of actin. Relative quantitation is expressed as fold-induction compared to control conditions (normal fibroblast group).

### 4.4. Statistical Analysis

All data are presented as mean ± standard error of the mean (SEM) and were analyzed using a paired *t*-test or one-way analysis of variance; *p* < 0.05 was considered statistically significant. SPSS version 19.0 (SPSS Inc., Chicago, IL, USA) was used for all statistical analyses.

## 5. Conclusions

Our study demonstrated that HMGB1 induces the activation of fibroblasts via activating the RAGE-MAPK and NF-κB interaction signaling pathways. Further study is needed to elucidate the mechanisms of ECM accumulation with exogenous HMGB1 administration and its action on EMT-mediated canonical signaling in wound repair. We expect that further knowledge of the relationship of skin fibrosis to HMGB1 may help to develop a new clinical approach to treat and prevent keloids and hypertrophic scars.

## Abbreviations

AKT	Protein kinase B
CXCL12	C-X-C motif chemokine 12
CXCR4	Chemokine Receptor type 4
ECM	Extracellular matrix
EMT	Epithelial-mesenchymal transition
ERK	Extracellular signal-regulated kinases
GA	Glycyrrhizic acid
HMGB1	High-mobility group box 1
MAPK	Mitogen-activated protein kinase
NF-κB	Nuclear factor kappa-light-chain-enhancer of activated B cells
PBS	Phosphate-buffered saline
PI3K	Phosphatidylinositol-4,5-bisphosphate 3-kinase
RAGE	Receptor for advanced glycation end product
TGF-β	Transforming growth factor-β
TLR	Toll-like receptor
